# T-Type Calcium Channels: A Potential Novel Target in Melanoma

**DOI:** 10.3390/cancers12020391

**Published:** 2020-02-08

**Authors:** Carla Barceló, Pol Sisó, Oscar Maiques, Inés de la Rosa, Rosa M. Martí, Anna Macià

**Affiliations:** 1 Oncologic Pathology Group, University of Lleida, IRBLleida, 25198 Lleida, Spain; carla_barcelo@medicina.udl.cat (C.B.); polsiso95@gmail.com (P.S.); inesrosazurera@gmail.com (I.d.l.R.); 2 Centre for Cancer and Inflammation, Barts Cancer Institute, Queen Mary University of London, London EC1M 6BQ, UK; o.m.carlos@qmul.ac.uk; 3 Department of Dermatology, Hospital Universitari Arnau de Vilanova, University of Lleida, IRBLleida, 25198 Lleida, Spain; marti@medicina.udl.cat; 4 Centre of Biomedical Research on Cancer (CIBERONC), Instituto de Salud Carlos III (ISCIII), 28029 Madrid, Spain

**Keywords:** T-type calcium channels (TTCCs), melanoma, BRAF^V600E^, therapy, resistance

## Abstract

T-type calcium channels (TTCCs) are overexpressed in several cancers. In this review, we summarize the recent advances and new insights into TTCC biology, tumor progression, and prognosis biomarker and therapeutic potential in the melanoma field. We describe a novel correlation between the Cav3.1 isoform and the increased basal autophagy in BRAF^V600E^-mutant melanomas and after acquired resistance to BRAF inhibitors. Indeed, TTCC blockers reduce melanoma cell viability and migration/invasion in vitro and tumor growth in mice xenografts in both BRAF-inhibitor-sensitive and -resistant scenarios. These studies open a new, promising therapeutic approach for disseminated melanoma and improved treatment in BRAFi relapsed melanomas, but further validation and clinical trials are needed for it to become a real therapeutic option.

## 1. Introduction

Calcium signaling displays important intracellular messages engaging multiple pathways and contributing to cellular homeostasis through calcium ions. Calcium is a second messenger that is involved in physiological functions such as viability, apoptosis, motility, exocytosis and endocytosis, and gene expression, and plays an important role in tumor growth [1,2,3,4]. Calcium channels support the entry of calcium in favor of concentration gradients across the plasma membrane into the cell, promoting a calcium influx [5]. These channels can be classified into selective and non-selective calcium channels. Non-selective calcium ion channels are mostly ligand-gated ion channels that open in response to a ligand and mediate cation flux. Additionally, store-operated calcium channels (SOCs) and voltage-gated calcium channels (VGCCs) are considered the two main kinds of selective calcium channels that mediate calcium influx in response to the depletion of intracellular calcium stores [6].

VGCCs are the most selective channels for calcium ions. VGCCs are heteromultimers assembled by a main α1-subunit and three auxiliary subunits, α2-δ, β, and γ, which regulate the expression and biophysical properties of the channels [7]. The α1-subunit is the largest one (190–250 kDa) contributing to the pore architecture and potential change sensor, in addition to being the main channel regulator, which determines the type of VGCC [8,9]. The α1-subunit of VGCCs is composed of four domains, which have six transmembrane segments (S1–S6) each [10]. The β subunits (β1–4) are cytosolic proteins that regulate current density, control channel activation/inactivation kinetics, and shift the voltage dependence and activation in the hyperpolarized direction. On the other side, the γ subunits (γ1–8) can have an inhibitory effect on calcium currents and can alter the activation/inactivation kinetics of the calcium channels [7,11,12,13,14].

VGCCs are activated and inactivated by plasma membrane depolarization and can be classified into three different families based on sequence homology and functional properties: (1) high-voltage-activated L-type channels (Cav1), (2) high-voltage-activated P/Q-type, N-type, and R-type channels (Cav2), and (3) low-voltage-activated T-type channels (Cav3). Three different genes that encode T-type calcium channels (TTCCs) have been described, including *CACNA1G*, *CACNA1H*, and *CACNA1I*, which encode for the main α-pore forming subunits Cav3.1, Cav3.2, and Cav3.3, respectively [15]. The diverse isoforms are differentiating due to their electrophysiological and pharmacological properties (Table 1). TTCCs are the only calcium low-voltage channels described to date, due to the fact that they require small depolarization to open. Further, they have a window current, as they can open but not inactivate completely, resulting in significant calcium entry at membrane potentials near rest [8,16,17].

## 2. T-Type Calcium Cannels in Cancer

VGCCs have been reported as promising candidates in targeted-cancer therapy [23]. Among VGCCs, TTCCs have been suggested as potential targets because of their upregulation in diverse tumor cells. Indeed, they are involved into the progression and prognosis of various cancers [14,24,25].

To date, it has been suggested that TTCC expression and isoforms are heterogeneous, depending on the stage of tumor development and cancer type. TTCCs, the Cav3.1 and Cav3.2 isoforms in particular, have been found to be overexpressed in prostate [26,27], breast [28,29,30], ovarian [31,32], colon [33] and esophageal [34] cancers, retinoblastoma [29], glioblastoma [35], hepatocellular carcinoma [36], and melanoma [18]. The Cav3.3 isoform has been reported to be increased in ovarian and esophageal cancer, hepatocellular carcinoma, and melanoma [14]. Consequently, the pharmacological inhibition of channels function or the molecular knockdown of TTCC expression is able to decrease proliferation and cell viability and increase apoptosis and arrest cells in the G1 phase in specific cancer types [14].

In silico data also supports this hypothesis. For instance, a microarray database (Oncomine) and bioinformatic analysis, performed by Phan and colleagues, indicated that both the up- and downregulation of TTCCs could be involved in a cancer’s molecular signature [23]. Such a study supports the complexity of calcium signaling, which needs further investigation in order to strongly associate VGCC expression within cancer progression. In addition, the aberrant hypermethylation of the *CACNA1G* gene (Cav3.1) can modulate TTCC expression. It has been found in various human primary tumors (colorectal, pancreatic, hepatic, and gastric cancer, as well as in acute myeloid leukemia), causing a drastic reduction in Cav3.1 levels. The inactivation of *CACNA1G* in particular neoplasms suggests that it may play a role as a tumor suppressor gene in such tumors [37,38,39,40].

## 3. Expression of T-type Calcium Channels in Melanoma

Cutaneous melanoma is a malignant skin cancer that arises from transformed melanocytes de novo or from congenital or acquired melanocytic nevi [41]. Melanoma is the most dangerous form of skin cancer, and its incidence is steadily increasing worldwide. Since melanoma can be diagnosed in young and middle-aged adults [42,43], it causes unbalanced mortality in that population, being responsible of one of the highest rates of loss of potential life for adult-onset cancers [44]. Melanoma cells have a high ability of local invasion and metastasis, even when arising from very small-volume tumors [45]. Once in advanced stages, the prognosis of melanoma is still poor, despite being the subject of intense research groups and numerous clinical trials that have increased therapeutic options, such as certain targeted and immunotherapies [46].

Available evidence indicates that calcium signaling plays an important role in melanoma cell viability and motility [47,48]. In 2012, it was reported for the first time that TTCCs were highly expressed in melanoma cells compared with melanocytes. Likewise, it was shown that TTCC expression was functional using calcium imaging techniques, which measure changes in the concentration of intracellular calcium flux through the membrane-permeable fluorescent dye Fura-2, which has the ability to bind to calcium [18].

**Figure 1:**
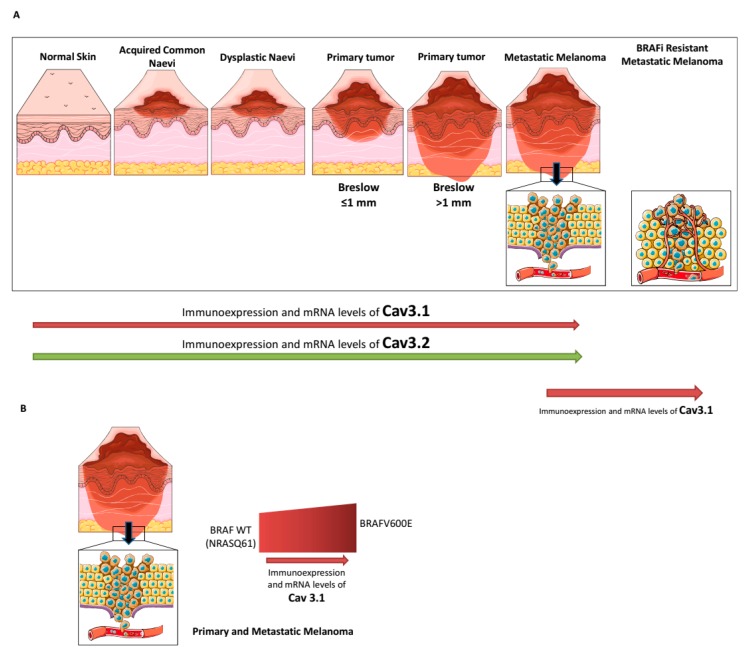
Cav3.1 and Cav3.2 T-type calcium channel (TTCC) expression during the progression of melanoma and depending on genetic profile. (**A**) Increased expression of Cav3.1 and Cav3.2 during melanoma progression (Clark model) and after acquisition of resistance to BRAF inhibitors (the last in the case of BRAF^V600E^ melanoma). (**B**) Expression of Cav3.1 in primary and metastatic melanoma depending on genetic profile (BRAF^V600E^ vs. non-BRAF-mutant melanoma).

Afterwards, using human biopsies, Maiques and colleagues were able to study the expression of TTCCs, comparing normal skin, melanocytic nevi, and melanoma tumors, with the aim of establishing a correlation between tumor progression and disease-free survival. By immunohistochemistry techniques, they described a progressive increase in the expression of TTCCs from normal skin to common nevi, dysplastic nevi, and primary and metastatic melanoma samples, but with differences in the distribution of TTCC isoforms (Table 1; Figure 1A). Particularly, Cav3.2 expression was significantly higher in metastatic melanoma than in primary melanoma, while Cav3.1 expression was increased in all samples of melanoma (both primary and metastatic). Moreover, a positive correlation was indicated between Breslow thickness-an important clinicopathological prognostic factor in primary melanomas-and the expression of TTCCs (Cav3.1 and Cav3.2) (Figure 1A) [21]. Additionally, the same study analyzed the presence of Cav3.1 and Cav3.2 isoforms in the four main primary cutaneous melanoma subtypes (superficial spreading malignant melanoma (SSMM), nodular melanoma (NM), acral lentiginous melanoma (ALM), and lentigo maligna melanoma (LMM)). Whilst the expression of Cav3.1 was not significantly different between the primary melanoma subtypes, the expression of Cav3.2 was higher in NM and ALM, suggesting a possible contribution of Breslow thickness [21].

It is well known that the most prevalent genetic hallmark of cutaneous melanoma is the presence of BRAF (BRAFV600E/K, 40–50%) or NRAS (NRASQ61, 20%) mutations, which are mutually exclusive [49]. Recently, the expression of TTCCs was investigated in different melanoma cell lines and human biopsies according to their genetic profile [20]. Melanoma cell lines that harbored BRAF^V600E^ mutation showed higher mRNA levels of the Cav3.1 and Cav3.3 isoforms compared with melanoma cells with NRAS mutation, whereas no significant differences were observed regarding Cav3.2 mRNA levels. Extending it to clinical settings, a cohort of primary and metastatic melanoma human biopsies bearing BRAF^V600E/K^ gene mutation showed a higher immunoexpression of Cav3.1 compared with a BRAF wild-type melanoma cohort (Table 1; Figure 1B) [20].

## 4. The Role of T-Type Calcium Channels in Melanoma

The concentration and tight regulation of calcium is essential for the communication of the extracellular medium, with different cellular compartments involved in calcium homeostasis during processes such as cell cycle, proliferation, apoptosis, and migration. TTCCs can regulate a variety of calcium-dependent cellular processes to support malignant growth, including proliferation, motility, survival, and differentiation.

### 4.1. Cell Proliferation and Apoptosis

**Figure 2:**
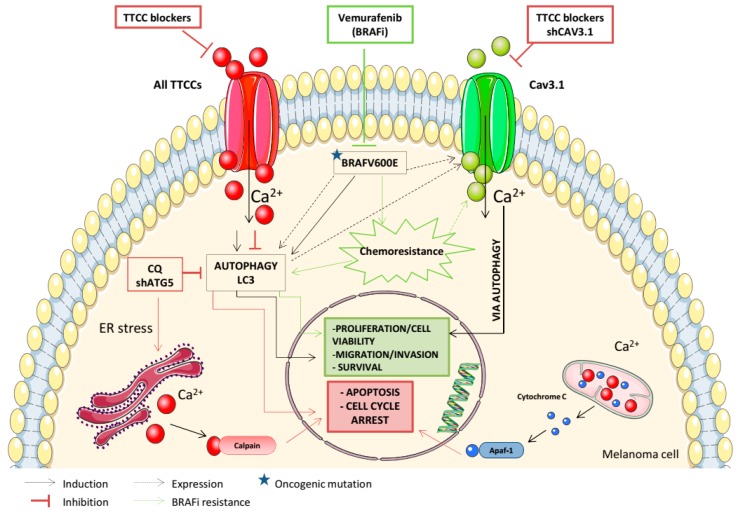
TTCCs play a significant role in cellular proliferation, apoptosis, migration, autophagy, and acquisition of BRAFi resistance in melanoma. The figure represents the relationships identified in different studies between TTCC expression and autophagy, and the effects of TTCC blockers in melanoma cells.

Cell proliferation is a complex mechanism orchestrated by several proteins and might be regulated by calcium signaling in different parts of the cell. TTCCs seem to be especially suitable for promoting cell cycle progression due to its rapid activation after weak depolarization. This feature allows for transient increases in cytosolic calcium in non-excitable cells that seem to favor mitotic progression by the direct binding of calcium to intracellular effectors such as calmodulin (CaM). VGCCs also participate in the progression of cancer by generating calcium wave oscillations, such as those provided by TTCCs, which favor the progression of the cell cycle [3]. Initial results showed that TTCC expression plays a crucial role in melanoma cell viability and in the induction of cell cycle progression (Figure 2). TTCC knockdown experiments of Cav3.1 and Cav3.2 genes have induced cell cycle arrest at the G1 and S phases. Specifically, the Cav3.1 isoform has been associated with slow cycling and upregulation under environmentally stressful conditions, such as hypoxia. It has also been shown that Cav3.2 expression correlates with high proliferation rates in melanoma cells [18]. In the same way, by immunodetection of different proteins involved in the progression and development of melanoma, it was determined that the Cav3.2 isoform was associated with Glut1 expression-a marker of hypoxia-as well as with cyclin D1 and Ki-67-two markers of proliferation [21].

Consequently, the inhibition of TTCCs affects their functionality; therefore, the cellular functions involved are compromised. Mibefradil was the first selective pharmacological TTCC inhibitor approved by the FDA; it was marketed by Roche as Posicor^®^ for the treatment of hypertension, but was later withdrawn due to drug-drug interactions [50]. However, Mibefradil has been recently re-approved, under the condition of “orphan medication,” so that its efficacy for the treatment of pancreatic cancer, glioblastoma multiforme, and ovarian cancer can be investigated (clinicaltrials.gov). Similarly, Pimozide-an antipsychotic drug-is also a strong inhibitor of TTCCs that inhibits tumor cell proliferation and decreases cell migration in hepatocellular carcinoma [51], prostate cancer [52], breast cancer [53], and melanoma [54].

In a follow-up study, Das and colleagues showed that TTCC blockers of clinical use (Mibefradil and Pimozide) had a dual effect on cell viability. They clearly decreased BrdU incorporation in melanoma cells compared with untreated cells, and a halt in cell proliferation was indicated, but a remarkable increase in apoptotic cell death was also observed (an increase in propidium iodide (PI) staining cells and the percentage of cells in the sub-G1 phase) (Figure 2). Further, it was determined that the apoptotic death of melanoma cells was partially dependent on caspase cascade activation, through the appearance of a cleavage fragment of effector caspase-3 after TTCC blocker treatment [19]. An in-depth analysis of the death process induced by TTCC blockers revealed that the apoptotic pathway was preceded by endoplasmic reticulum (ER) stress and the subsequent inhibition of basal macroautophagy, which is constitutively active in melanoma cells (Table 1) [19,21,55,56]. TTCCs have been suggested to couple the calcium influx to ER calcium storage [57]. It has been shown that TTCC blockers induce ER stress with an upregulation of unfolded protein response (UPR) markers (chaperone GRP78, transcription factor XBP-1, and GADD153) as an anti-tumoral strategy in melanoma cells [19].

### 4.2. Autophagy

Autophagy is a catabolic process that helps to maintain cellular homeostasis, so that cells can degrade and recycle damaged organelles and proteins through catabolism [58]. The crosstalk between calcium and autophagy has a central role in cellular homeostasis and survival during several physiologic and pathologic conditions [59]. Autophagy is constitutively induced in melanoma cells and is a housekeeping process involved in tumor progression and melanoma metastasis [19,56,60,61].

Our group was the first to show that the Cav3.1 isoform is upregulated in both melanoma cell lines and biopsies from BRAF^V600E^-mutant melanomas, which was accompanied by increased levels of LC3II protein-important for the autophagic process-compared with NRAS-mutant melanomas [20]. In line with this, we described a significant correlation between the presence of BRAF^V600E^-mutant protein with Cav3.1 expression and a positive link to LC3II protein in a cohort of primary and metastatic melanomas (Table 1; Figure 2) [21]. These results reinforce the basal autophagy present in melanoma, which is enhanced in BRAF^V600E^-mutant melanoma cells and related to the upregulation of the Cav3.1 isoform.

TTCC blockers inhibit autophagy and induce cell death in all melanoma cells, regardless of the specific mutation present [19,20]. Recently, our group performed in vivo xenograft mouse models with subcutaneous injections of melanoma cell lines, with a further administration of Mibefradil daily via oral gavage for 2 w. We showed that TTCC blocker treatment significantly reduces tumor growth and induces apoptosis via an autophagy blockade in xenograft melanoma models (Table 1) [22].

Mitophagy is an essential process that maintains mitochondrial quality and number, thus limiting cellular degeneration. Along with apoptosis, mitophagy participates in cellular fate decisions by eliminating damaged mitochondria. A variety of mitochondrial parameters (structure, membrane potential, own machinery, etc.) are important regulators of the mitochondrial capacity for calcium uptake due to their close apposition with the ER [62]. Mitochondrial calcium is rapidly balanced by an equivalent calcium outflow from the organelle, which depends on the exchange of sodium, but only up to a cytosolic concentration called the “mitochondrial set point.” TTCCs have to satisfy both the maintenance of a sustained influx of calcium into the cell and the direct part of this calcium towards the mitochondria. Gouriou and colleagues have shown that cytosolic calcium elevations were paralleled by mitochondrial calcium elevations, which were also increased by TTCCs overexpression [63].

Autophagy-mediated degradation of ER fragments or ER-phagy contributes to the removal of aberrant protein products from the lumen or ER membrane. The ER acts as a dynamic intracellular calcium reservoir, controlling cytosolic calcium levels [64]. It has been shown that disruptions in the cellular energy levels, the redox state, or calcium concentrations reduce the protein folding capacity of the ER, and lead to the accumulation and aggregation of unfolded proteins, resulting in ER stress [65].

### 4.3. Migration and Invasion

Cell migration is a process whereby cancer cells escape the primary tumor and invade other tissues to develop metastases. Maiques and co-workers assessed the effect of TTCC blockers on the migration and invasion of melanoma cells using wound-healing, single-cell, and transwell assays [66]. We showed that TTCC blockers reduce the migration rates and invasive potential of BRAF^V600E^-mutant melanoma cells due to autophagy inhibition, with no significant effect in NRAS-mutant melanoma cells (Table 1; Figure 2) [20,66]. Melanoma cell invasion and subsequent metastasis are hallmarks of melanoma dissemination [67]. Snail1 is a transcription factor that induces epithelial-mesenchymal transition (EMT) [68], and it has been shown to be crucial during melanoma cell motility and invasiveness [20,69,70]. Moreover, Snail1 expression was higher in BRAF^V600E^-mutant melanomas compared with BRAF wild-type melanoma cells and biopsies, and its expression decreases upon an autophagy blockade by TTCC blockers. Consequently, we stated that Snail1 could be essential during a metastatic cascade of melanomas with BRAF mutation [20]. All the effects produced by pharmacological blockers of TTCCs related with the induction of apoptosis, the blockade of autophagy, and the reduction of migration/invasion processes in melanoma cells were mimicked by TTCC gene silencing (Figure 2) [19,20].

### 4.4. Tumor Progression and Prognostic Marker

Several studies emphasize the expression of TTCCs not only as a marker of tumor progression, but also as a prognostic marker. Indeed, in silico analysis of the database from The Cancer Genome Atlas (TCGA) indicated that disease-free survival (DFS) and overall survival (OS) were inversely correlated with the increased expression of Cav3.2, and DFS was also inversely correlated with overexpression of Cav3.1 (Figure 2). All results indicate that the expression of these two TTCC isoforms correlates with the tumor progression of melanoma and that their overexpression in the primary tumor may be an indicator of poor prognosis [21].

## 5. T-Type Calcium Channels and BRAF Inhibitor Resistance in Melanoma

A high frequency of activation of several mechanisms in cancer cells underlying the development of resistance under pharmacological treatment (chemotherapy, target therapy or immunotherapeutic agents) has been widely described. As mentioned, a high percentage of melanoma cases (40–60%) present BRAF^V600^ mutation (V600E or V600K) [71]. Vemurafenib was the first BRAF^V600E^ drug inhibitor (BRAFi) approved for the treatment of advanced melanoma. However, progressive disease after a short period of administration was one of the main issues (common to other BRAF inhibitors, such as Dabrafenib), leading to the development of secondary resistance [72]. A number of molecular mechanisms underlying this resistant phenotype have already been elucidated, by reactivation of the MAPK and/or the PI3K-Akt pathways [73,74,75,76]. Therefore, a deeper characterization of resistance mechanisms to BRAFi remains essential for determining new-generation therapeutic strategies [77,78,79].

An increasing number of studies have shown a clear link between calcium channel expression and sensitivity to therapeutic drugs. Many calcium channels are involved in quimioresistance acquisition in a large variety of cancers [80]. To date, in addition to our group results, there are no studies about TTCCs and melanoma treatment resistance acquisition. Our team is the first to propose that overexpression of TTCC Cav3.1 could be a key mechanism in the acquisition of BRAFi resistance in melanoma cells. In this context, we set out to study the modulation of TTCCs and/or the inhibition of autophagy as a possible therapy for Vemurafenib-resistant melanoma. In fact, our group recently demonstrated that chronic exposure to Vemurafenib-induced drug resistance in BRAF^V600E^-mutant melanoma cells was due to a Vemurafenib-promoting autophagic process, a mechanism that contributes to BRAFi resistance in melanoma cells [22,81,82,83]. In addition, Barceló and colleagues showed that both Vemurafenib-resistant BRAF^V600E^-mutant melanoma cells and biopsies from human melanoma relapsing under BRAFi expressed higher levels of the Cav3.1 isoform and the LC3 protein compared with their parental Vemurafenib-sensitive cell line or pre-treatment melanoma tumors, respectively (Table 1; Figure 1A and Figure 2). In silico analysis reinforced this observation, indicating that Cav3.1 enrichment and enhanced basal autophagy influence the arising intrinsic mechanism of BRAFi resistance in melanoma. Furthermore, in the same study, we determined that a TTCC blocker (Mibefradil) decreases cell viability and induces apoptosis as well as impairs migration and invasion rates in Vemurafenib-resistant melanoma cells due to autophagy inhibition (Table 1; Figure 2). In addition, oral administration of Mibefradil in SCID mice models reduced tumor growth in resistant melanoma cells and induced apoptosis via a blockade of autophagy [22]. These results suggest that the inhibition of autophagy, induced indirectly through TTCC blockers, could be a therapeutic tool to drive apoptosis and to reduce migration and invasion rates in BRAFi melanoma-resistant cells. It therefore could be a new therapy against the secondary resistance of melanoma arising after chronic treatment with Vemurafenib.

## 6. T-type Calcium Channels as a Therapeutic Target in Melanoma

Identifying TTCC expression in tumors and characterizing their functionality may introduce alternative treatments, particularly for patients in which the tumor relapses after standard therapies.

At present, it is possible to arrest cancer cell proliferation and induce cell death by inhibiting TTCCs on a wide range of cancer cells. Conversely, the use of TTCC pharmacological blockers has proven effective in reducing the viability of tumor cells grown in vitro [19,20,22,30,31,84] and in preclinical tumor growth mice models [22,30,31,85,86]. There are a variety of agents that could affect TTCCs with varying degrees of specificity. As mentioned, the TTCC blocker, Mibefradil, is an FDA “orphan drug” approved for its efficacy to treat ovarian cancer (2007), pancreatic cancer (2008), and glioblastoma multiforme (2009) (https://www.accessdata.fda.gov/scripts/opdlisting/oopd/listResult.cfm). Mibefradil has been recognized as a proliferative inhibitor in many different cell lines, including mononuclear blood cells [87], leukemia [88], glioblastoma [89], retinoblastoma [90], and tumor cells of the pituitary gland [91]. Apart from its anti-proliferative properties, Mibefradil has been shown to affect cell motility and the invasive properties of fibrosarcoma [92].

Melanomas are highly heterogeneous due to their mutational and epigenetic profiles and, when disseminated, poorly respond to chemotherapy and radiotherapy regimens, so diverse specific targets involved in melanoma progression are under evaluation. Therefore, the development of innovative strategies remains critical for increasing available anticancer therapies for melanoma. A number of publications reporting TTCC expression (especially Cav3.1 and Cav3.2) in melanoma are now available. These describe the involvement of TTCCs in melanoma progression and as a prognostic biomarker, Cav3.1 upregulation in BRAF^V600E^-mutant melanomas, and further involvement in the acquisition of resistant mechanisms to conventional treatments (Figure 2) [19,20,21,22]. It has been shown that pharmacological inhibitors of TTCCs induce a cell cycle arrest, induce a caspase-dependent apoptosis, and reduce tumor growth preceded by the activation of ER stress and the subsequent inhibition of the autophagic flux, constitutively activated in melanoma cells [19,22]. Indeed, Mibefradil treatment induces cell death and reduces the migration/invasion rates in BRAF^V600E^-mutant melanoma cells due to autophagy blockade, as a possible emerging therapeutic strategy against melanoma progression (Table 1; Figure 2) [20]. In accordance, the inhibition of enhanced autophagy by TTCC blockers reduce tumor aggressiveness in recurrent Vemurafenib-resistant melanomas, decrease Cav3.1 expression, and avoid the acquisition of resistance to BRAFi treatment in BRAF^V600E^-mutant melanoma cells [22,93]. For all described, TTCC blockers could be targetable to deregulate autophagy and may offer a new mechanism to combat melanoma progression and therapeutic resistance to conventional anticancer drugs.

## 7. Conclusions

TTCC blockers reduce the viability and tumor growth of melanoma cells, providing a new target therapy for melanoma treatment. Identifying an enhanced TTCC expression in melanoma and characterizing their function may introduce additional treatment alternatives, especially for patients who do not respond to standard therapies or develop resistant tumors. The expression of Cav3.1, with a positive correlation with autophagy biomarkers related to melanoma progression, prognosis, and resistance acquisition to BRAFi, can lead to the development of new therapeutic strategies by which TTCCs are blocked in order to deal with the development and emergence of metastatic melanoma. Thus, new biochemical compounds that block TTCCs could become valuable partners to impede mid- and long-term melanoma progression.

## Figures and Tables

**Table 1:** T-type calcium channel (TTCC) expression and cellular function in melanoma. ND: not determined.

Cellular Functions of TTCCs	Cav3.1	Cav3.2	Cav3.3
Molecular Information	Human: 2377aa, O43497, chr. 17q22, CACNA1G	Human: 2353aa, O95180, chr. 16p13.3, CACNA1H	Human: 2251aa, AAM67414, chr. 22q13.1, CACNA1I
Threshold mV	−70	−70	−70
mRNA	+	+	+
Protein	IHC	IHC	ND
Functional Channels	+	+	+
Prognosis in Melanoma	Negative	Negative	ND
Expression Levels	↑ mRNA expression in melanoma cell lines and biopsies compared with melanocytes [18]	↑ mRNA expression in melanoma cell lines and biopsies compared with melanocytes [18]	↑ mRNA expression in melanoma cell lines and biopsies compared with melanocytes [18]
	Hypoxia ↑ mRNA expression (M16, JG, M28) [19]	Hypoxia ↑ mRNA expression [19]	↑ mRNA expression in BRAFV600E melanoma [20]
	↑ immunoexpression from normal skin to naevi to melanoma biopsies (tumor progression) [21]	↑ immunoexpression from normal skin to naevi to melanoma biopsies (tumor progression) [21]	
	↑ immunoexpression in primary melanoma tumors with Breslow thickness >1 mm [21]	↑ immunoexpression in primary melanoma tumors with Breslow thickness >1 mm [21]	
	↑ immunoexpression positively correlates with BRAFV600E protein expression and LC3 [21]	↑ immunoexpression positively correlates with Glut1 and Ki-67 [21]	
	↑ mRNA expression and immunoexpression in BRAFV600E melanoma [20]		
	↑ mRNA expression and immunoexpression in BRAF inhibitor-resistant melanoma [22]		
	↑ mRNA expression during acquisition of Vemurafenib resistance in melanoma [22]		
	↓ mRNA expression after TTCC blocker treatment in sensitive and Vemurafenib-resistant melanoma [22]		
Cellular function	TTCC blocker or molecular knockdown of Cav3.1 expression arrest cells in G1 phase and decreases cell viability in melanoma cells [18,19,22]	TTCC blocker or molecular knockdown of Cav3.2 expression arrest cells in G1 phase and decreases cell viability in melanoma cells [18,19,22]	TTCC blocker arrests cells in G1 phase and decreases cell viability in melanoma cells [18,19,22]
	TTCC blocker or molecular knockdown of Cav3.1 expression impairs migration/invasion rates in BRAFV600E-mutant melanoma cells [20]	TTCC blocker or molecular knockdown of Cav3.2 expression impairs migration/invasion rates in BRAFV600E-mutant melanoma cells [20]	TTCC blocker impairs migration/invasion rates in BRAFV600E-mutant melanoma cells [20]
	TTCC blocker reduces tumor growth in vivo [5]	TTCC blocker reduces cell viability and inhibit migration/invasion rates in Vemurafenib-resistant melanoma cells [20]	TTCC blocker reduces cell viability and inhibit migration/invasion rates in Vemurafenib-resistant melanoma cells [20]
	TTCC blocker reduce cell viability and inhibit migration/invasion rates in Vemurafenib-resistant melanoma cells [20]	TTCC blocker reduce tumor growth in vivo [22]	TTCC blocker reduce tumor growth in vivo [22]
Relative to Autophagy	TTCC blocker or molecular knockdown of Cav3.1 blocks autophagy flux in all melanoma cells [19,20,22]	TTCC blocker or molecular knockdown of Cav3.2 blocks autophagy flux in all melanoma cells [19,20,22]	TTCC blockade inhibits autophagy flux in all melanoma cells [19,20,22]
	TTCC blocker inhibit autophagy flux in Vemurafenib-resistant melanoma cells [22]	TTCC blocker inhibit autophagy flux in Vemurafenib-resistant melanoma cells [22]	TTCC blocker inhibit autophagy flux in Vemurafenib-resistant melanoma cells [22]
